# CUL4A overexpression as an independent adverse prognosticator in intrahepatic cholangiocarcinoma

**DOI:** 10.1186/s12885-017-3389-z

**Published:** 2017-06-02

**Authors:** Gong -Kai Huang, Ting-Ting Liu, Shao-Wen Weng, Huey-Ling You, Yu-Ching Wei, Chang-Han Chen, Hock-Liew Eng, Wan-Ting Huang

**Affiliations:** 1 Department of Laboratory Medicine, Kaohsiung Chang Gung Memorial Hospital and Chang Gung University College of Medicine, Kaohsiung, Taiwan; 2 Department of Pathology, Kaohsiung Chang Gung Memorial Hospital and Chang Gung University College of Medicine, Kaohsiung, Taiwan; 3 Department of Internal Medicine, Kaohsiung Chang Gung Memorial Hospital and Chang Gung University College of Medicine, Kaohsiung, Taiwan; 4 Department of Pathology Kaohsiung Municipal Ta-Tung Hospital, Kaohsiung Medical University Hospital, Kaohsiung, Taiwan; 5 Department of Medical Laboratory Sciences and Biotechnology, Fooyin University, Kaohsiung, Taiwan; 6 Institute for Translational Research in Biomedicine, Kaohsiung Chang Gung Memorial Hospital, Kaohsiung, Taiwan; 7 Department of Applied Chemistry, National Chi Nan University, Nantou, Taiwan; 8 Center for Infectious Disease and Cancer Research, Kaohsiung Medical University, Kaohsiung, Taiwan; 9 Department of Laboratory Medicine, Kaohsiung Medical Center, Chang Gung Memorial Hospital, 123, Ta-pei Road, Niao-Sung District, Kaohsiung City, Taiwan

**Keywords:** CUL4A, Intrahepatic cholangiocarcinoma, Immunohistochemical study, Disease-free survival, Migration and invasion assays

## Abstract

**Background:**

CUL4A has been known for its oncogenic properties in various human cancers. However, its role in intrahepatic cholangiocarcinoma (iCCA) has not been explored.

**Methods:**

We retrospectively investigated 105 iCCA cases from a single medical institution. Tissue microarrays were used for immunohistochemical analysis of CUL4A expression. *CUL4A* expression vectors were introduced in cell lines. Cell migration and invasion assays were used to compare the mobility potential of iCCA cells under basal conditions and after manipulation. Then we evaluated the effects of CUL4A on the cell growth by proliferation assay, and further checked the susceptibility to cisplatin in iCCA cells with or without CUL4A overexpression.

**Results:**

CUL4A overexpression was detected in 34 cases (32.4%). Patients with CUL4A-overexpressing tumors exhibited shortened disease-free survival (mean, 27.7 versus 90.4 months; *P* = 0.011). In the multivariate analysis model, CUL4A overexpression was shown to be an independent unfavorable predictor for disease-free survival (*P* = 0.045). Moreover, stably transfected CUL4A-overexpressing iCCA cell lines displayed an increased mobility potential and enhanced cell growth without impact on susceptibility to cisplatin.

**Conclusions:**

Our data demonstrate that overexpression of CUL4A plays an oncogenic role in iCCA and adversely affects disease-free survival. Thus, it may prove to be a powerful prognostic factor and a potential therapeutic target.

## Background

*CUL4A* (*Cullin 4A*) is located at the 13q34 chromosomal loci; it contains 20 exons and encodes an 87-kDa protein [1]. It belongs to the cullin family and functions as a component of a multifunctional ubiquitin-protein ligase E3 complex that is called CRL (cullin-RING ubiquitin ligase). CRL mediates the process of ubiquitylation (also called ubiquitination) of a wide range of substrates involved in normal cellular physiology. CUL4A has an arc-shaped helical N-terminal domain that binds to a specific adaptor or substrate receptor [2]. The targeted substrates are involved in diverse cellular processes, including cell cycle progression, signal transduction, genetic transduction, tumor suppression, the DNA damage response, and chromatin remodeling [1]. Thus, any deregulation of CUL4A expression and/or alteration of its function are expected to have a profound effect on cellular physiology.

Unsurprisingly, there are increasing number of studies focused on the relationship between CUL4A and tumorigenesis, since deregulation of the cell cycle and genome instability, i.e., two of the most common features of cancer cells, may result from abnormal CUL4A expression [3]. Primary breast cancer was the first type of carcinoma in which amplification and overexpression of the *CUL4A* gene was detected, back in 1998 [4]. Since then, similar observations have been made in hepatocellular carcinomas [5], malignant pleural mesotheliomas [6], and prostate cancers [7]. Overexpression of CUL4A may lead to the proliferation, progression, and metastasis of cancer [8, 9].

Intrahepatic cholangiocarcinoma (iCCA) is a relatively rare and aggressive form of cancer, accounting for 5–15% of all primary liver cancers worldwide [10]. The high mortality rate and poor prognosis of iCCA are associated with early invasion, widespread metastasis, and the lack of an effective therapy [11]. In a recent cohort study of 86 iCCA patients, we discovered that recurrent amplification at 13q14 was an independent adverse prognosticator, with *CUL4A* being one of the amplification targets [12]. However, we did not explore the relationship between the levels of CUL4A expression and the clinicopathologic features of iCCA. In the present study, we aimed to examine the frequency of CUL4A overexpression and whether this aberration correlates with iCCA disease progression. To this end, we first collected 105 iCCA cases from a single institution and used formalin-fixed, paraffin-embedded tissues to assemble tissue microarrays for immunohistochemical (IHC) staining. Results showed that CUL4A protein levels positively correlated with clinicopathologic features. Furthermore, experiments with two stably CUL4-overexpressing iCCA cell lines showed that CUL4 increases the cell mobility potential.

## Methods

### Case selection

We selected 105 iCCA cases from the patient base of the Department of Pathology, Chang Gung Memorial Hospital at Kaohsiung, Taiwan. Samples had been collected in the period from 1989 to 2012. Medical records of the respective patients were available and were carefully reviewed. Survival time was defined as the period between the date of diagnosis and the date of death or the patient’s last follow-up. The hematoxylin- and eosin-stained sections obtained at the time of diagnosis and repeats were reviewed. We adopted The American Joint Committee on Cancer (AJCC) 7th edition staging system for iCCA. The study was approved by the Institutional Review Board of Chang Gung Medical Foundation, in accordance with the Helsinki Declaration (IRB201600720B0 and IRB 103-6997B).

### Tissue microarrays and immunohistochemical analysis

A total of 105 formalin-fixed, paraffin-embedded iCCA tissue samples were used for tissue microarray construction. From each tumor specimen, quadruplicate tissue cores with diameters of 1.0 mm were punched out with a Beecher tissue microarrayer (Beecher Instruments, Silver Spring, MD, USA). Serial 5 μm thick tissue sections were cut from microarrays for IHC study, which was performed with a Leica Bond-III automated immunostainer (Leica Biosystems, Wetzlar, Germany) using anti-CUL4A as the primary antibody (cat. no EPR3198, rabbit monoclonal, 1:100; Abcam, Cambridge, MA, USA). The slides were evaluated by two pathologists (GKH and TTL) blind to clinicopathologic data. Tumors containing a minimum of two or more analyzable cores were scored. Whole sections were stained for IHC analysis in cases with non-informative tissue cores (no tumor cells present, or fewer than 2 analyzable cores). Breast carcinomas and normal bile ducts were used as positive and negative controls, respectively. The percentages of tumor cells with detectable nuclear immunoreactivity for CUL4A were recorded using a 5% increment. The labeling intensity was given a score from 0 to 3, corresponding to non-detectable, weak, moderate and strong staining, respectively. An expression index was defined as the product of the percentage of immunoreactive positive tumor cells and the labeling intensity. Obviously, the index could range from 0 to 300, with 300 corresponding to all (100%) tumor cells displaying strong (3) staining. The scores of multiple cores from the same patient were averaged to obtain a mean expression index. After testing a series of cutoff values, we decided to construe the CUL4A protein as overexpressed when the expression index was equal to or higher than 50.

### Cell lines and stable transfection

The iCCA cell lines, SSP-25 (Resource No. RBRC-RCB 1293, Lot No. 003) and RBE (Resource No. RBRC-RCB 1292, Lot No. 003), were purchased from the Riken BRC Cell Bank (Koyadai, Japan), respectively. Tumor cell lines were cultured in Gibco Roswell Park Memorial Institute (RPMI) medium (Thermo Fisher Scientific, Waltham, MA, USA) as described previously [12]. Cells were transfected with the pCMV-CUL4A entry vector using the Invitrogen lipofectamin 2000 reagent (Thermo Fisher Scientific), according to the manufacturer’s instructions. Cells were selected by growth in complete medium containing Neomycin (Sigma, St. Louis, MO, USA). Total cell lysates were analyzed for CUL4A protein levels by western blotting.

### Western blot analysis

Western blotting was performed using a sodium dodecyl sulfate-polyacrylamide gel electrophoresis system as described previously [12]. Immunoblotting was performed by incubation at 4 °C with antibodies against CUL4A (1:1000; CST) and β-actin (1:2000, Santa Cruz Biotechnology) overnight. Blots were then washed and incubated with a 1:2000 dilution of horseradish peroxidase (HRP)-conjugated secondary antibody (Jackson, West Grove, PA, USA), followed by three washes with Tris-buffered saline-containing Tween 20. Pierce Enhanced chemiluminescent HRP substrate (Thermo Fisher) was used for detection according to the manufacturer’s instructions.

### Cell migration and invasion assays

Cell migration and invasion were assessed as described previously [12]. Briefly, total 200 μL of cell suspension was added to the top wells of the chamber with 8-μm pores, which were coated with 0.1 mL of diluted Matrigel Matrix coating solution from Corning (Corning, NY, USA) for the invasion assay, or left uncoated for the migration assay. The average cell mobility was determined by counting three random high-powered fields at ×100. Three independent experiments were performed for both invasion and migration assays.

### Proliferation assay

Cell viability was determined by the XTT (tetrazolium hydroxide salt) assay according to the manufacturer’s instructions (Roche, Basel, Switzerland). Cells (1.0 × 10^4^ cells/well) were plated into 96-well culture plates for three different time periods (24 h, 48 h and 72 h). Then the XTT reagent was added with an incubation of 4 h, the spectrophotometric absorbance of the resulting solution was measured at 570 nm with a reference of 650 nm in a Sunrise microplate reader (Tecan, Männedorf, Switzerland). Each experiment was carried out in triplicate and performed at least thrice separately.

### Assessment of therapeutic drug effect on cell growth

Cells (1.0 × 10^4^ cells/well) were plated into 96-well culture plates for a 24-h incubation period prior to cisplatin (Sigma-Aldrich, catalog #P4394, St. Louis, MO) treatment. Then medium was replaced with serum-free media containing varying concentrations of cisplatin (0, 1, 2.5, 5 and 10 µM) and incubated for 24 h and 48 h. The cell viability was determined by the XTT assay as described previously.

### Statistical analysis

All statistical analyses were performed using the Statistical Product and Service Solutions (SPSS) v17.0 software (SPSS Inc., Chicago, IL, USA). Fisher’s exact test and chi-square test were used to determine the statistical significance level for the association between CUL4A expression and histopathological variables. Overall Survival (OS) was defined as the time between diagnosis and death from any cause, whereas Disease-Free Survival (DFS) was measured as the period from surgery to recurrence in the liver or distant metastasis. The Kaplan-Meier method was used for univariate survival analysis, whereas the difference between survival curves was tested by a log-rank test. In a stepwise backward fashion, parameters with *P* < 0.05 at the univariate level were entered into a Cox regression model to analyze their relative prognostic importance. However, vascular invasion and tumor growth patterns of the 7th AJCC staging system were not introduced into the multivariate analyses. Comparisons between different groups were performed using the Student’s *t*-test. For all analyses, two-sided tests of significance were used with *P* < 0.05 considered significant.

## Results

### Clinicopathologic data

The cohort consisted of 58 males (55.2%) and 47 females (44.8%) with a median age of 58 years (range, 30–84; mean, 58 years). Fifty-two patients had undergone lymphadenectomy, of whom 12 (23%) had developed lymph node metastasis. Forty-seven (44.8%) of the 105 patients exhibited local recurrence at a median follow-up period of 8.9 months (range, 0.2–84.5). Forty-two (40%) patients exhibited distant metastasis at a median follow-up period of 5.1 months (range, 0.9–44.7). The median follow-up period was 28.6 months (range, 2.7–176.9). The overall 3- and 5-year survival rates were 44.8% and 28.6%, respectively.

### Correlation between CUL4A expression and clinicopathologic variables

**Fig. 1:**
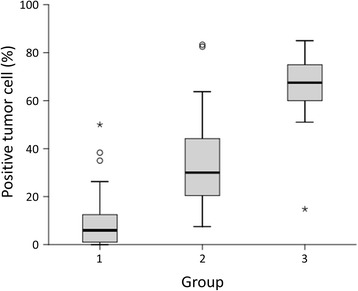
Differential expression of CUL4A protein in intrahepatic cholangiocarcinoma. (Group 1: the score of the average intensity was lower than or equal to 1; group 2: the score was higher than 1 and lower than 2; group 3: the score was higher than or equal to 2)

**Fig. 2:**
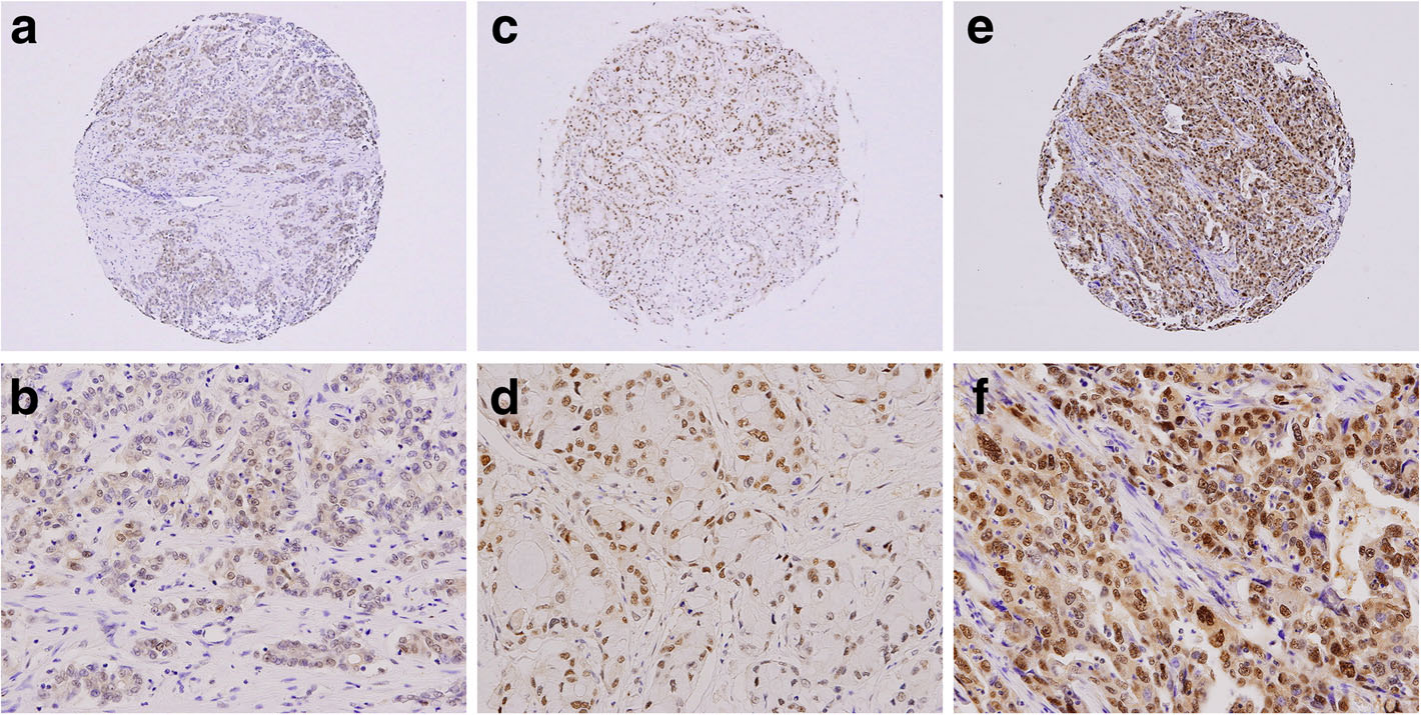
Representative photographs of CUL4A immunostaining in intrahepatic cholangiocarcinoma. Panels **a**, **c**, and **e** represent TMA cores at magnification 40×; **b**, **d**, and **f** represent selected areas from **a**, **c**, and **e** at higher magnification (200×). Expression indexes were calculated by multiplying the percentage of positive tumor cells by the average intensity. (**a** and **b**) Weak staining (1+) with 10% positive tumor cells. (**c** and **d**) Moderate staining (2+) with 60% positive tumor cells. (**e** and **f**) Strong staining (3+) with 75% positive tumor cells

**Fig. 3:**
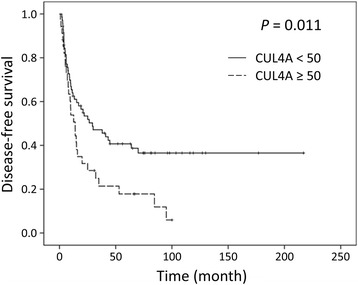
Kaplan-Meier survival curves for patients categorized by CUL4A expression index. Statistical significance was observed between groups. (CUL4A < 50: CUL4A expression index lower than 50; CUL4A ≥ 50: CUL4A expression index higher than or equal to 50)

Kaplan-Meier univariate survival analysis revealed that the following clinicopathologic variables were significantly associated with reduced survival (Table 1): infiltrative tumor growth pattern, multiple tumor number, larger tumor size, inadequate resection margin, vascular invasion, neural invasion, and advanced tumor stage. Immunoexpression of CUL4A protein could be successfully interpreted in 105 cases. The average intensity positively correlated with the number of immunoreactive positive tumor cells (Fig. 1). A mean expression index equal to or higher than 50, which, as mentioned earlier, was defined as the cutoff value separating normal expression from overexpression, was observed in 34 cases (32.4%) (Fig. 2). No correlation between CUL4A overexpression and histopathological parameters was observed. However, patients with tumors overexpressing CUL4A showed significantly shortened DFS (mean, 27.7 versus 90.4 months; *P* = 0.011; Fig. 3). Multivariate Cox proportional hazards regression analysis was used to derive risk estimates related to disease-free survival for CUL4A overexpression and clinicopathologic factors (Table 2). In addition to tumor size, resection margin, and tumor stage, CUL4A overexpression was shown to be an independent unfavorable DFS predictor (*P* = 0.045).

**Table 1:** Results of univariate long-rank analysis of prognostic factors for overall survival and disease-free survival

Parameters	No. of patients	Overall survival		Disease-free survival	
		No. of events	*P* value	No. of events	*P* value
Age, years					
≤ 60	54	31	0.452	36	0.757
> 60	51	33		34	
Gender					
Male	58	39	0.392	38	0.538
Female	47	25		32	
Gross pattern					
MF	62	33	0.004^a^	40	0.142
MF + PI	42	31		29	
Tumor N					
Solitary	87	50	0.021^a^	54	0.003^a^
Multiple	16	12		15	
Tumor size					
≤ 5 cm	52	32	0.084	27	< 0.001^a^
> 5 cm	46	29		38	
Margin					
≤ 1 cm	66	45	0.025^a^	49	0.011^a^
> 1 cm	28	15		17	
Necrosis					
≤ 10%	77	45	0.134	49	0.104
> 10%	28	19		21	
VI					
No	64	37	0.122	35	0.001^a^
Yes	41	27		35	
NI					
No	67	35	< 0.001^a^	39	0.003^a^
Yes	38	29		31	
H grade					
I	29	19	0.776	17	0.398
II + III	76	45		53	
pT					
T1	34	16	0.008^a^	18	0.005^a^
T2 - T4	67	47		50	
LN					
No	40	23	0.198	26	0.353
Yes	12	8		9	
Stage					
I	30	14	0.006^a^	16	0.009^a^
II + III + IV	71	49		52	
CUL4A					
< 50	71	42	0.245	42	0.011^a^
≥ 50	34	22		28	

*M* mass-forming type, *PI* periductal infiltrating type, *N* number, *VI* vascular invasion, *NI* neural invasion, *H* histology, *pT* tumor stage, *LN* lymph node metastasis

^a^Statistically significant

**Table 2:** Independent predictive factors of disease-free survival by multivariate analysis

Variable	Hazard Ratio	95% CI	*P*
Tumor size ≤5 cm vs > 5 cm	1.986	1.19 to 3.32	0.009
Resection margin ≤1 cm vs > 1 cm	1.809	1.01 to 3.24	0.046
Stage I vs II & III & IV	2.190	1.22 to 3.92	0.008
CUL4A expression index <50 vs ≥ 50	1.688	1.01 to 2.82	0.045

### Overexpressing *CUL4A* in iCCA cell lines alters their migratory and invasive capacities *in vitro*

**Fig. 4:**
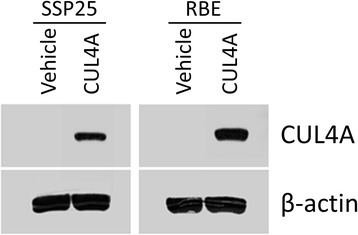
CUL4A overexpression in SSP25 and RBE cells. Expression levels of CUL4A were analyzed by Western blot

**Fig. 5:**
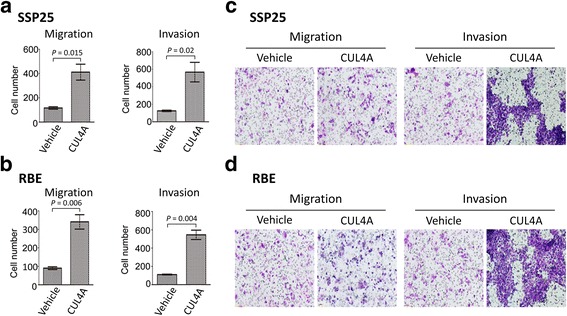
CUL4A promotes migration and invasion of iCCA cells. SSP25-CUL4A (**a** and **c**), RBE-CUL4A (**b** and **d**), and control vehicle cells were subjected to Transwell migration and Matrigel invasion assays. Quantification of migrated cells through the membrane and invaded cells through Matrigel of each cell line are shown as cell numbers. All results are from three independent experiments

To determine the effects of CUL4A on the mobility of cancer cells, we established two stably CUL4A-overexpressing cell lines, designated as SSP-25-CUL4A and RBE-CUL4A. Western blot analyses verified the upregulation of CUL4A expression (Fig. 4). SSP-25-CUL4A cell line displayed higher numbers of both migratory cells and invasive cells compared to the vehicle control cells (*P* = 0.015 and *P* = 0.02, respectively, Fig. 5a and c). Similarly, RBE-CUL4A cell line also revealed significantly greater migratory potential for migration and invasion (*P* = 0.006 and *P* = 0.004, respectively, Fig. 5b and d).

### Impact of CUL4A overexpression on cell growth and susceptibility to cisplatin

**Fig. 6:**
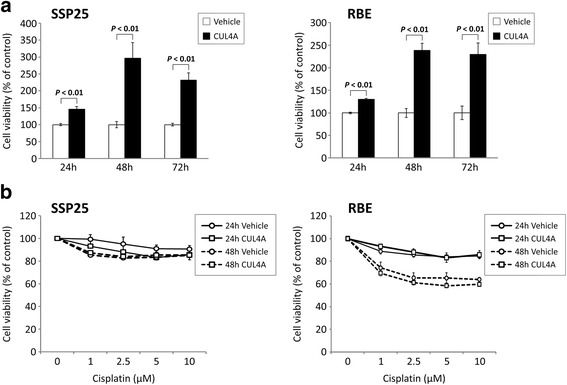
The effects of CUL4A on cell growth and susceptibility to cisplatin in iCCA cells. Cell viability was assessed by XTT assay at 24, 48, and 72 h (**a**). The results are presented as percentage viability of the vehicle control cells. Then we treated iCCA cells with cisplatin at different concentrations for the indicated time periods (**b**). The results are presented as percentage viability of untreated control. Data represent means ± standard deviation from three experiments

We then examined the influence of CUL4A overexpression on cell growth. Cell viability of the two stably CUL4A-overexpressing cell lines was evaluated at the three time points. Both SSP-25-CUL4A and RBE-CUL4A cell lines exhibited an enhancing effect on cell growth, which showed statistically significant differences when compared to the vehicle control cells (Fig. 6a). To study the effect of CUL4A on the susceptibility to chemotherapeutic drugs, we further checked the cell viability of iCCA cell lines after treatment with increasing concentrations of cisplatin at the two time points. The CUL4A over-expressing iCCA cell lines revealed similar trends of shifting of the cell viability as compared with vehicle control cells (Fig. 6b). SSP-25 cell line was less susceptible to cisplatin treatment than RBE cell line and the susceptibility of the both cell lines were not influenced by CUL4A overexpression.

## Discussion

In this study, we characterized CUL4A overexpression as an adverse prognostic factor of DFS in iCCA. In addition to tumor size, surgical resection margin and tumor stage, CUL4A is an independent factor associated with DFS in iCCA patients. Overexpressing *CUL4A* in iCCA cell lines enhanced their mobility potential, with respect to both migration and invasion capacity. The CUL4A-overexpressing iCCA cells were more proliferative but revealed no changes of the susceptibility to cisplatin. Taken together, these results clearly suggest that overexpression of CUL4A can serve as an adverse prognostic factor mainly through promoting tumor progression with increased cell motility. To our knowledge, this is the first study to elucidate the oncogenic role of CUL4A by immunohistochemistry in iCCA tumor samples.

Whether or not tumor size affects postoperative survival in iCCA remains a highly disputed topic. After analyzing 598 patients from the Surveillance Epidemiology and End Result (SEER) database, Nathan et al. came to the conclusion that tumor size failed to predict survival in patients with iCCA [13]. As a result, the tumor cutoff size of 5 cm was omitted from the AJCC/UICC staging schema. In our study, however, an iCCA tumor size >5 cm was an independent prognostic factor of shorter disease-free survival. Other studies also provided data supporting that tumor size has an effect on the clinical outcome of iCCA. Sakamoto et al. analyzed 419 patients who underwent surgical resection and found that overall survival was best stratified using a tumor size cutoff value of 2 cm, even though the multivariate analysis failed to identify tumor size as a significant prognostic factor [14]. Similarly, Uenishi et al. reported that iCCA patients having tumors with size ≤2 cm had a markedly favorable prognosis [15]. With respect to the different tumor size cutoff values that were clinically significant, Gil et al. supported that a tumor of size >4.0 cm along with lymph node metastasis and the presence of multiple tumors were significant predictors of iCCA recurrence [16]. Both Ali et al. and Hwang et al. reported that a tumor with size >5 cm was a risk factor associated with tumor recurrence and poorer iCCA patient survival [17, 18]. The association of tumor size with being an adverse prognostic factor of the clinical outcome of iCCA may be due to its correlation with increased incidence of vascular invasion and higher tumor grade [19]. The aforementioned studies add to the conflict on the validity and accuracy of tumor size as a prognostic indicator for being included in the 7th AJCC/UICC staging system introduced in 2010. Because of the difficulty in diagnosing small-sized iCCAs clinically, the exact effect of tumor size on survival is still unknown and will require further study involving higher numbers of patients.

In recent years, accumulating research data have demonstrated that CUL4A is overexpressed in multiple human cancers and contributes to tumor progression and metastasis, resulting in poorer survival rates of cancer patients. Hung et al. reported that CUL4A protein is overexpressed in malignant pleural mesothelioma [6]. Schindl et al. revealed that high expression of CUL4 is associated with a significantly lower overall and disease-free survival in node-negative breast cancer [20]. Melchor et al. reported that 13q34 amplification is related to tumor progression of basal-like breast cancers by inducing overexpression of CUL4A and TFDP1 [8]. In addition, prostatic cancers harboring highly expressed CUL4A were found to have poorer overall survival, while knockdown of CUL4A inhibits cancer cell growth *in vitro* and *in vivo* [7]. CUL4A protein overexpression was also identified as an adverse prognostic factor in epithelial ovarian cancers [21].

After the proto-oncogenic properties of CUL4A had been elucidated, efforts to investigate *CUL4A* copy number alternations were made to clarify the relationships between chromosomal aberrations and protein expression levels. Studies utilizing comparative genomic hybridization (CGH) found recurrent 13q14 amplification, of which *CUL4A* may be a target, in various types of tumors, including esophageal squamous cell carcinoma [22], adrenocortical carcinoma [23], hepatocellular carcinoma [5], and childhood medulloblastoma [24]. Recently, we reported the detection of 13q14 amplification in iCCA. *CUL4A* was one of the targets of the amplification, with the number of copies correlating with protein expression [12].

The underlying biochemical mechanism through which CUL4A regulates tumor development and progression has been widely discussed. There is increasing evidence indicating that CUL4A plays an important role in cell cycle regulation by degrading or upregulating cell cycle proteins (cyclins), cyclin-dependent kinases (CDKs), and cyclin-dependent kinase inhibitors (CDKIs). CUL4A is associated with MDM2-mediated proteolysis of p53 through the ubiquitin-proteasome pathway [25]. CRL4, which is the CUL4A-containing CRL complex, mediates proteolysis of p21 and p27, which facilitate S-phase progression by inhibiting the activity of cyclin-E/CDK2 and cyclin-A/CDK2, or cyclin-E/CDK2 alone [26]. In addition, the CRL4 complex has been found responsible for inactivation and/or degradation of p73 [27], p27 [28–30], the p12 subunit of DNA polymerase δ (Pol δ) [31], and the histone methyltransferase Set8 [32]. Therefore, CUL4A may deregulate cell cycle, damage DNA repair, and lead to genome instability, resulting in tumorigenesis.

Epigenetic modification, such as histone methylation, is another of the diverse mechanisms through which CUL4A affects tumor progression. In epithelial cancers, mounting evidence suggests the crucial role of epithelial–mesenchymal transition (EMT) in tumor invasion and metastasis. EMT is essential for tumor cells’ ability to disseminate from their original tissues to seed new tumors in distant sites. CUL4A could be a factor influencing EMT. Evidence for this was provided by the study of Wang et al. who reported that CUL4A transcriptionally activates ZEB1 (Zinc finger E-box-binding homeobox 1) expression via increasing the levels of H3K4 (histone H3 lysine 4) trimethylation [9], resulting in the subsequent decrease in the levels of epithelial markers (E-cadherin and α-catenin) and the increase in the abundance of mesenchymal markers (N-cadherin, fibronectin, and vimentin) in tumor cells, which are characteristic of EMT. The correlation between EMT and patient outcomes in iCCA was reported by Gu et al. [33]. Loss of β-catenin combined with aberrant expression of vimentin or fibronectin was associated with poor histological differentiation and overall and disease-free survival. The mechanism through which CUL4A regulates ZEB1 expression may also affect EGFR expression. In a study on patients with non-small cell lung cancer (NSCLC), Wang et al. found that CUL4A overexpression significantly increased the levels of both EGFR transcript and protein through CUL4A-mediated recruitment of H3K4met3 to the EGFR promoter [34]. The subsequent activation of the EGFR-AKT pathway leads to cancer cell proliferation, inhibits apoptosis, and enhances chemotherapy resistance. The authors also suggested that directly targeting CUL4A with the purpose of disrupting this oncogenic signaling pathway might lead to tumor-inhibitory effects. Other tumor-related signal-transduction pathways are also modulated by CUL4A expression. In 2008, a study speculated that overexpression of CUL4A may promote the degradation of the tumor suppressor TSC2 (tuberous sclerosis 2) protein, resulting in the upregulation of the mTOR (mammalian target of rapamycin) pathway [35]. Another study suggested a synergistic effect between CUL4A overexpression and the activation of the H-RAS (v-Ha-ras Harvey rat sarcoma viral oncogene) pathway in the tumorigenesis of basal-like breast cancers. Conversely, *in vitro* and *in vivo* results have showed that downregulation of CUL4A leads to the inhibition of breast cancer growth [36].

In our previous cohort study of 86 iCCA patients, we discovered that *CUL4A* was one of the amplification targets as an adverse prognosticator, and knockdown of *CUL4A* gene dramatically reduced migratory and invasive capacities of iCCA cells *in vitro* [12]. In current study, we further found CUL4A-overexpressing cell lines behaved more aggressively featuring increased cellular proliferation and greater migratory potential, with respect to both migration and invasion capacity. These results indicate CUL4A would be required for aggressive iCCA cell lines to be invasive and migratory. However, the CUL4A over-expressing iCCA cell lines revealed no significant differences in response to treatment with cisplatin when compared with vehicle control cells. That may suggest overexpression of CUL4A can serve as an adverse prognostic factor mainly through signals promoting cell growth, migration and invasion in iCCA.

Because of the important role of CUL4A in the ubiquitin-proteasome system, which plays a role in diverse cellular processes, development of drugs targeting the system is a promising and vital field in cancer therapy. Bortezomib was the first proteasome inhibitor approved by the US Food and Drug Administration for the treatment of multiple myeloma and lymphoma [37, 38]. However, its many side effects limit its clinical use. MLN4924, a newly developed selective inhibitor of NEDD8 (neural precursor cell expressed developmentally downregulated 8)-activating enzyme, can disrupt CRL-mediated protein turnover leading to apoptotic death in human cancer cells, while its use caused fewer side effects [39]. In recent years, the potential therapeutic value of directly targeting CUL4A was also put forth by researchers, e.g., inhibition of CUL4A ubiquitin ligase was found to prevent UV-associated skin cancer and premature aging [40]. With increasing knowledge, development of iCCA anti-cancer therapy targeting CUL4A can be expected in the near future.

## Conclusions

This study suggests that CUL4A may be a useful biomarker to predict disease progression in iCCA. Overexpression of CUL4A is correlated with tumor recurrence and promotes tumor progression. In order for this protein to be established as a powerful prognostic factor and potential therapeutic target, subsequent studies are required for clarifying the mechanisms underlying CUL4A-induced migration and invasion by iCCA.

## Abbreviations

AJCC: American Joint Committee on Cancer
AKT: v-Akt Murine Thymoma Viral Oncogene, a serine/threonine kinase also known as protein kinase B (PKB)
CDK: Cyclin-dependent kinase
CDKI: Cyclin-dependent kinase inhibitor
CGH: Comparative genomic hybridization
CRL: Cullin-ring ubiquitin ligase
*CUL4A*: Gene name for Cullin 4A
CUL4A: Protein name for Cullin 4A
DFS: Disease-free survival
EGFR: Epidermal growth factor receptor
EMT: Epithelial–mesenchymal transition
H3K4: Histone H3 lysine 4
H3K4met3: Histone H3 lysine 4 trimethylation
H-RAS: v-Ha-ras Harvey rat sarcoma viral oncogene homolog
HRP: Horseradish peroxidase
iCCA: Intrahepatic cholangiocarcinoma
IHC: Immunohistochemistry
MDM2: Murine double minute 2
mTOR: Mammalian target of rapamycin
NEDD8: Neural precursor cell expressed developmentally down-regulated 8
NSCLC: Non-small cell lung cancer
OS: Overall survival
SEER: Surveillance Epidemiology and End Result database
TFDP1: Transcription factor Dp-1
TSC2: Tuberous sclerosis 2
UICC: Union for International Cancer Control
UV: Ultraviolet
ZEB1: Zinc finger E-box-binding homeobox 1
